# Oxidative Stress and Mitochondrial Dysfunction in Chronic Kidney Disease

**DOI:** 10.3390/cells12010088

**Published:** 2022-12-25

**Authors:** Hsin-Jung Ho, Hitoshi Shirakawa

**Affiliations:** 1 Faculty of Health Sciences, Hokkaido University, Sapporo 060-0812, Japan; 2 Laboratory of Nutrition, Graduate School of Agricultural Science, Tohoku University, Sendai 980-8572, Japan; 3 International Education and Research Center for Food Agricultural Immunology, Graduate School of Agricultural Science, Tohoku University, Sendai 980-8572, Japan

**Keywords:** oxidative stress, chronic kidney disease, mitochondrial homeostasis, mitochondrial turnover

## Abstract

The kidney contains many mitochondria that generate ATP to provide energy for cellular processes. Oxidative stress injury can be caused by impaired mitochondria with excessive levels of reactive oxygen species. Accumulating evidence has indicated a relationship between oxidative stress and kidney diseases, and revealed new insights into mitochondria-targeted therapeutics for renal injury. Improving mitochondrial homeostasis, increasing mitochondrial biogenesis, and balancing mitochondrial turnover has the potential to protect renal function against oxidative stress. Although there are some reviews that addressed this issue, the articles summarizing the relationship between mitochondria-targeted effects and the risk factors of renal failure are still few. In this review, we integrate recent studies on oxidative stress and mitochondrial function in kidney diseases, especially chronic kidney disease. We organized the causes and risk factors of oxidative stress in the kidneys based in their mitochondria-targeted effects. This review also listed the possible candidates for clinical therapeutics of kidney diseases by modulating mitochondrial function.

## 1. Introduction

The kidney is one of the most fuel-hungry organs in the human body, and the kidney and heart showed higher resting metabolic rates than the brain or other organs [1]. The kidney requires the second highest mitochondrial density and oxygen consumption after the heart to provide energy for a variety of cellular functions and processes, including removing waste products, reabsorbing nutrients, balancing the body’s fluids, and regulating blood pressure [2,3]. Oxidative stress injury can derive from impaired mitochondria with excessive levels of reactive oxygen species (ROS), because ROS are a byproduct of adenosine triphosphate (ATP) generation. ROS are produced in the cytoplasm, plasma membrane, endoplasmic reticulum, and mitochondria; the latter are the major site of ROS production [4,5]. Under normal conditions, mitochondrial ROS (mtROS) formation and release are regulated by the regenerative cycle ROS-induced ROS release. An imbalance in ROS-induced ROS release may trigger various cellular signaling pathways with the consequent onset of numerous pathologies, including cell death [6]. Evidence indicates that mtROS play a key role in the progression of several diseases, including kidney diseases, due to the high mitochondrial content in kidney tissue. Recent studies have suggested that dysregulation of mitochondrial homeostasis, alterations in bioenergetics, and organelle stress cause renal diseases [7]. Mitochondrial dysfunction leads to decreased ATP levels and increased ROS levels, resulting in renal dysfunction. Therefore, the increase in ATP production and mitochondrial antioxidant defense, as well as the maintenance of mitochondrial homeostasis in the kidney, are considered therapeutic targets for oxidative stress-related renal injury [8,9,10].

Nephron loss, inflammation, and endothelial injury with vascular rarefaction are considered the causes for developing progressive acute kidney injury and chronic kidney disease (CKD). Despite the limitations of multi-organ interactions, cell experiments are available for investigating cell-specific mechanistic pathways. Animal models of CKD are considered as a useful tool to validate novel treatment interventions which mimic human CKD [11]. To understand the novel mitochondrial-targeted interventions in the fields of CKD and the relationship between mitochondrial and renal function, we organized articles related to oxidative stress and mitochondrial dysfunction in kidney diseases, especially CKD, published in the last decade. This review pointed out the mitochondria-targeted tools and summarized the relationship between mitochondrial function and the risk factors of CKD. The content is divided into three parts: 1) the relationship between oxidative stress and mitochondria in kidney diseases, 2) mitochondria-targeted therapeutics for kidney diseases, and 3) causes and risk factors of oxidative stress-related mitochondrial dysfunction in CKD.

## 2. Relationship between Oxidative Stress and Mitochondria in Kidney Diseases

**Figure 1:**
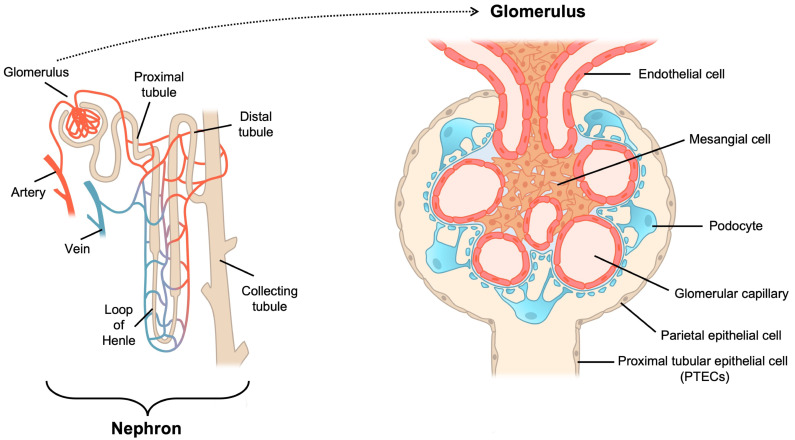
Nephron structure in the kidney. The nephron is the functional unit of the kidney. The glomerulus is the filtering unit located at the beginning of a nephron. Glomerular cells comprise endothelial cells, mesangial cells, podocytes and epithelial cells. Oxidative stress likely causes dysfunction of these cells, leading to renal damage.

The nephron is a functional renal unit. It consists of endothelial cells, mesangial cells, podocytes, parietal epithelial cells, tubular epithelial cells, and collecting duct cells (Figure 1). Mitochondrial injury increases oxidative stress in these cells and may cause renal damage such as tubulointerstitial injury, glomerular hypertrophy, podocytopathy, and nephrotic syndromes, and may even increase the risk of complications that frequently lead to mortality [12,13,14].

Patients present with symptoms during CKD, including irreversible nephron number decrease, inflammation, fibrosis and reduced regenerative capacity [15]. CKD is defined as renal damage persisting for more than three months. CKD is staged based on the estimated glomerular filtration rate and albuminuria, with conditions ranging from asymptomatic to end-stage renal disease (ESRD). Various complications of CKD include atherosclerosis and hypertension. Higher rates of cardiovascular morbidity and mortality have been observed in patients with CKD [15]. These elevated rates could be associated with an increase in renal oxidative stress, which may result from an imbalance in ROS levels and antioxidant defense [16]. A recent review summarized the main sources of ROS in tissues and pointed out that mitochondria and the NADPH oxidase (NOX) family are the major sources of endogenous ROS in the kidney [17]. The increased mtROS levels and/or NOX activity as a result of disruptions in cellular redox homeostasis may be an important contributor to CKD progression.

In vitro, the renal cells have been used to assess the progression of CKD according to their own mechanisms in the kidney [12,13]. However, the limited results can also be interpreted by specific responses in the single cell type without the crosstalk among the cells in CKD. In vivo studies provide opportunities in several animal models that mimic human CKD [18,19,20,21,22,23,24]. Five-sixth nephrectomy (5/6Nx), unilateral ureteral obstruction (UUO), and adenine diet are widely used models for investigating the mechanistic pathways involved in CKD progression to mimic and predict responses in human CKD [18,19]. The surgical approaches 5/6Nx and UUO mimic the renal failure after loss of renal mass in humans, which are useful to assess glomerulosclerosis and tubulointerstitial fibrosis caused by reducing renal blood flow and glomerular filtration rate [18]. At a pharmacological concentration of adenine, the poor solubility of adenine derivatives in urine can accumulate in renal tubules or interstitial tissues and lead to renal injury [19]. Adenine also acts on renal tubules as a signaling molecule, and causes nephrogenic diabetes insipidus with salt wasting and renal fluid loss [19,20]. The adenine diet model features rapid-onset renal failure with extensive fibrosis, tubular atrophy, crystal formation, and marked vascular calcification [21]. Oxidative stress and apoptosis of vascular smooth muscle cells are key to vascular calcification in patients with CKD and progression to ESRD [21]. Moreover, renal failure can be accompanied by increased levels of serum creatinine, blood urea nitrogen, urinary protein, inflammatory cytokines, profibrotic factors, lipid peroxidation, and mitochondrial dysfunction in the adenine diet [20], 5/6Nx [22], and UUO models [23,24].

**Figure 2:**
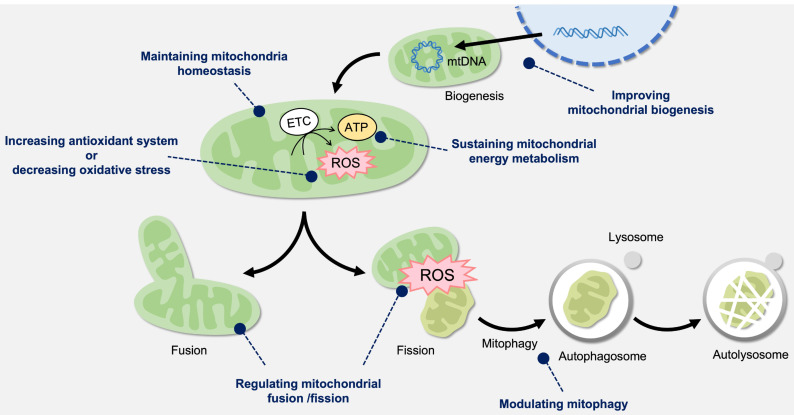
Mitochondria-targeted therapeutics for oxidative damage. Mitochondria produce energy via the oxidative phosphorylation process in the electron transport chain (ETC) present in the mitochondrial membrane. Mitochondrial membrane potential (MMP) plays a key role in mitochondrial homeostasis. To maintain mitochondrial function, fusion reduces stress and enhances integrity by sharing the contents as a form of complementation. Fission segregates the damaged region of mitochondria. Mitochondrial biogenesis is the process that regulates the number of healthy mitochondria, resulting in increased ATP production. Mitophagy is the selective process for removing the dysfunctional mitochondria. The complicated processes can control mitochondrial quality and cell metabolism, which may also provide possible therapeutic targets for oxidative damage.

Numerous studies have reported that oxidative stress-related kidney diseases can also cause mitochondrial dysfunction. We highlighted the potential of mitochondria as therapeutic targets for renal injury and provided valuable insights into clinical strategies. As shown in Figure 2, mitochondria-targeted therapeutics may be associated with the maintenance of mitochondrial homeostasis, increased mitochondrial antioxidant capability or decreased oxidative stress, sustained mitochondrial energy metabolism, improved mitochondrial biogenesis, regulation of mitochondrial fusion/fission, and modulation of mitophagy.

### 2.1. Mitochondrial Homeostasis

**Figure 3:**
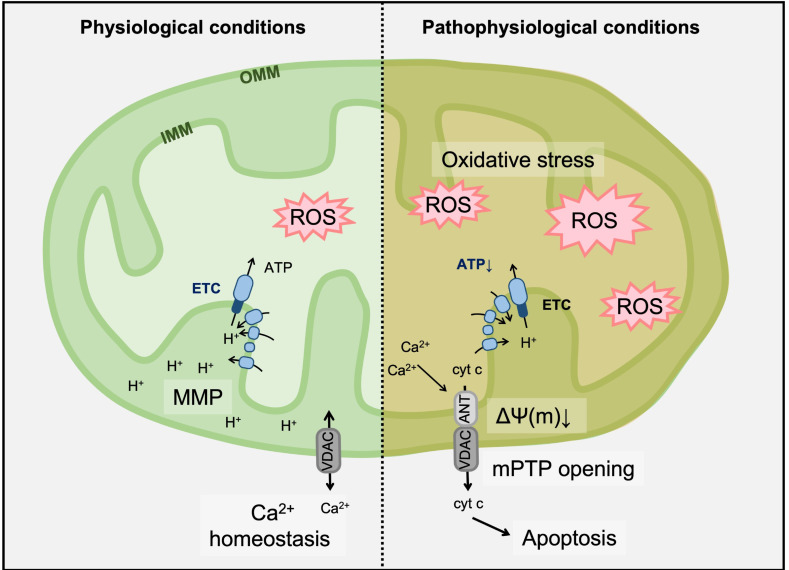
Regulation of mitochondrial permeability transition pore (mPTP) for maintaining mitochondria homeostasis. mPTP is a protein complex present between the inner mitochondrial membrane (IMM) and outer mitochondrial membrane (OMM). The mPTP is composed of a voltage-dependent anion channel (VDAC) and adenine nucleoside translocator protein (ANT) that act to regulate MMP for mitochondria homeostasis. Under pathophysiological conditions, excessive Ca^2+^ and ROS induce mPTP opening and cause mitochondrial-related apoptosis.

The opening of the mitochondrial permeability transition pore (mPTP) has an important physiological role in maintaining healthy mitochondrial homeostasis. Increased mitochondrial calcium ion (Ca^2+^) levels are important in mPTP opening. As shown in Figure 3, under normal physiological conditions, mPTP allows small substances to move into mitochondria and drive ATP synthase through oxidative phosphorylation (OXPHOS) to maintain mitochondrial membrane potential MMP (ΔΨ(m)). Conversely, in pathophysiological conditions, elevated oxidative stress or overloaded Ca^2+^ stimulates abnormal mPTP opening. Consequently, the passage of large substances and soluble substances causes MMP collapse, uncoupling of OXPHOS, and mitochondrial swelling. Additionally, the stimulated release of mitochondrial cytochrome c (cyt c) into the cytoplasm induces apoptotic cell death [25,26].

Sodium thiosulfate has been used to improve calcific uremic arteriopathy in dialysis patients [21]. Sodium thiosulfate maintains mitochondrial homeostasis via Ca^2+^ chelation and improves renal mitochondrial respiratory capacity by maintaining MMP and restoring electron transport chain (ETC) activity in an adenine-induced CKD model [27]. The substance 10-(6′-Ubiquinonyl)decyltriphenylphosphonium bromide (mitoquinone, MitoQ) is a small molecule. Notably, MitoQ can act as a mitochondria-targeted antioxidant that selectively accumulates in the mitochondria of several tissues, including the kidney. MitoQ decreased oxidative stress and cell death in renal tubular cells, rat and porcine kidneys against cold storage injury [28,29]. MitoQ can also protected renal tubular cells in diabetic nephropathy [30]. However, recent studies have suggested that MitoQ may have adverse effects. In these studies, MitoQ increased ROS production in various cancer cells, which was associated with decreases in MMP and mtDNA copy number [31,32]. A recent study further showed that MitoQ causes acute mitochondrial swelling and MMP depolarization in renal proximal tubular cells, suggesting that MitoQ should be used carefully as a clinical medicine in patients with renal or other diseases [33]. Together, the evidence suggested that mitochondrial homeostasis has been associated with renal function in CKD or diabetic nephropathy.

### 2.2. Mitochondrial Antioxidant System

**Figure 4:**
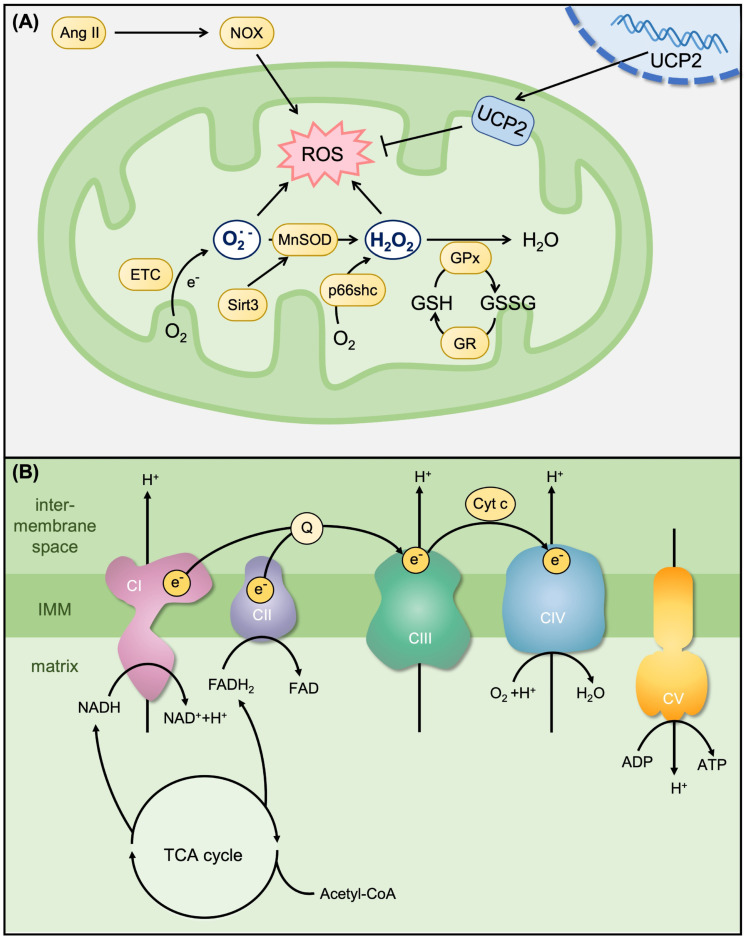
Mitochondrial antioxidant system and OXPHOS. (**A**) Mitochondrial antioxidants and ROS production. Mitochondria are the major source of superoxide (O_2_˙^−^) in aerobic organisms. ETC generate ATP and results in O_2_˙^−^ production, which is converted to hydrogen peroxide (H_2_O_2_) by manganese-superoxide dismutase (MnSOD), and then converted to H_2_O by glutathione peroxidase (GPx). Uncoupling protein-2 (UCP2) also plays a key role in the control of intracellular oxidative stress. (**B**) Mitochondrial ETC and OXPHOS. Electrons (e^−^) are transferred from reduced nicotinamide adenine dinucleotide (NADH) to oxidized form (NAD^+^) in complex I (CI) or from flavin adenine dinucleotide (FADH_2_)-containing enzymes complex II (CII) to reduce CoQ10 (Q). The electrons are then transferred to complex III (CIII, bc1 complex) and cyt c. Finally, H_2_ reacts with O_2_ to yield H_2_O in complex IV (CIV). Complex V (CV, ATP synthase) synthesizes ATP from ADP, which is driven from the proton (H^+^) gradient (produced by CI, CIII and CIV). In addition, the NADH and FADH_2_ for OXPHOS are produced by both fatty acid β-oxidation and the tricarboxylic acid (TCA) cycle. Acetyl-CoA is generated from fatty acids (β-oxidation), amino acids or pyruvate (glycolysis) for initial mitochondrial TCA cycle reactivation.

Mitochondrial ETC and other enzymes generate superoxide (O_2_˙^−^), which is converted to hydrogen peroxide (H_2_O_2_) by superoxide dismutase (SOD) [34,35]. H_2_O_2_ participates in various cellular signaling pathways, such as the regulation of mitochondrial antioxidant defenses (Figure 4A). To maintain cellular ROS balance, mitochondria possess their own antioxidant enzyme system to desensitize redox signals, including manganese SOD (MnSOD) and glutathione peroxidase [36]. However, excessive H_2_O_2_ bursts from the mitochondria to the cytosol induce oxidative stress and cause cell damage [34,35,36]. As mentioned above, both mitochondria and NOX are major sources of H_2_O_2_ generation [37]. NOX4 is the most abundantly expressed NOX isoform in the kidneys, and NOX4-derived ROS overproduction in mesangial, endothelial, and tubular cells has been associated with kidney disease related to diabetes and obesity [17,37]. In one study, upregulation of NOX4 by angiotensin II caused an increase in ROS generation in mesangial cells, whereas inhibition of Nox4 abolished the increase in ROS generation. Moreover, angiotensin II elicited an increase in mtROS levels. Overexpression of the mitochondrial antioxidative enzyme MnSOD prevented the effects of angiotensin II on mtROS production in mesangial cells [38]. Diphenyleneiodonium and dextromethorphan are NOX inhibitors [39,40]. Diphenyleneiodonium inhibits ROS production in H_2_O_2_-induced renal medullary interstitial cells and UUO-mediated renal injury [39]. Dextromethorphan attenuates vascular oxidative stress and decreases aortic calcification in rats [40]. These findings suggest that regulation of angiotensin II-mediated NOX4 activation and mtROS production may be a useful strategy to inhibit oxidative stress. However, both MnSOD and glutathione peroxidase can remove ROS, but uncoupling protein-2 (UCP2) is an anion transporter that also plays a key role in oxidative stress [41]. UCP2 activity is stimulated by ROS, which then acts through a negative feedback mechanism, decreases ROS levels, and protects cells from oxidative damage [41]. In the human kidney, UCP2 is mainly localized in the renal proximal tubular cells to maintain cell function [42]. In diabetic kidneys, overexpression of UCP2 is involved in regulating MMP to prevent excessive mitochondrial O_2_˙^−^ formation [43]. UCP2 knockdown resulted in a paradoxical increase in adenine nucleotide translocase-mediated uncoupling. However, uncoupling reportedly increased total O_2_ consumption and potentially amplifies existing renal hypoxia, thereby accelerating the development of diabetic nephropathy [43]. These findings suggest that mitochondrial uncoupling is associated with ROS levels and development of diabetes-induced renal injury. In addition, mitochondrial chaperones prohibitin [44] and Sirtuin 3 (Sirt3) [45] also regulate ROS generation and mitochondrial homeostasis, which are related to UCP2 activity. The inhibition of UCP2 upregulation resulted in the reduction of mtROS production and promotion of ATP generation in prohibitin-overexpressing H_2_O_2_-induced injury of proximal tubular cells [44].

Antioxidants may remove ROS or alter the antioxidant system, and also regulate mitochondrial bioenergetics and/or dynamics. In a study, mito-TEMPO ameliorated renal fibrosis by alleviating mitochondrial dysfunction through the Sirt3-MnSOD antioxidative pathway in 5/6Nx mice [46]. The accumulation of mtROS can contribute to microvascular dysfunction in CKD patients, suggesting that oxidative stress is the key to vascular calcification in patients with CKD [47]. As mentioned above, MitoQ can act on renal mitochondrial homeostasis with beneficial [28,29,30] or adverse effects [31,32,33]. MitoQ attenuates vascular calcification by suppressing oxidative stress in vascular smooth muscle cells through the nuclear factor erythroid 2-related factor 2 (Nrf2)-mediated antioxidant pathway [48]. However, a recent study suggested that the protective effect of MitoQ on vascular hemodynamics is a result of augmented cardiac function or a reduction in vascular resistance [49]. Although mitochondrial-targeted antioxidants showed potent properties for protecting mitochondrial function, the safety of antioxidants in CKD patients should be further investigated [28,29,30,31,32,33,48,49]. These data indicated that mitochondrial redox signaling and antioxidative defense play a key role in oxidative stress. Antioxidants such as mito-TEMPO [46] and MitoQ [48] showed benefits on the reduction of ROS via regulating the antioxidant pathway.

### 2.3. Sustaining Mitochondrial Energy Metabolism

Mitochondria are key players in the generation and regulation of cellular bioenergetics, producing ATP by OXPHOS in the IMM (Figure 4B). Mitochondrial dysfunction leads to a decrease in ATP production, alterations in cellular function and structure, and loss of renal function [50]. ATP production is decreased by multiple mechanisms, including an impaired tricrboxylic acid cycle, glycolysis, and fatty acid β-oxidation, which provide precursors for ATP generation [51,52]. Increasing evidence indicates that the transition to CKD is accompanied by persistent decreases in fatty acid β-oxidation with renal tubular cell atrophy but continued high expression of glycolytic enzymes [52]. Moreover, in Sirt3 deficient mice, the basal levels of ATP in the kidney were reportedly reduced by >50%. Mitochondria from these mice displayed selective inhibition of Complex I activity [53]. The findings suggest that Sirt3 not only regulates ROS generation [45] but is also an important regulator of Complex I activity and maintains ATP levels [53].

A time course study suggested that the 5/6Nx model features decreased mitochondrial β-oxidation at early time points, as well as impaired ATP production [54]. These alterations are linked to an early decrease in complex I and ATP synthase activities and to a further decrease in complex III activity [54]. The increased expression of NADH-ubiquinone oxidoreductase core subunit V1 (an enzyme in complex I) in the kidney improved the integrity of complex I and increased complex I activity to mitigate renal oxidative damage in UUO mice [24]. The evidence indicated that mitochondrial bioenergetics impairment is an early event in renal damage, whose persistence in time aggravates CKD development; however, improvement of mitochondrial energy metabolism can mitigate renal damage. Moreover, mitochondrial energy metabolism can be sustained by pirfenidone and fluorofenidone [55]. These are antifibrotic drugs with potential abilities to reduce ROS levels, maintain mitochondrial structure, increase ATP production, and increase mtDNA copy number in a complex Ι inhibitor, rotenone-treated renal proximal tubular cells. Pirfenidone and fluorofenidone also reportedly reduced oxidative stress by enhancing MnSOD and inhibiting intracellular ROS generation, suggesting that the antioxidant effects occurred at least partially via the mitochondrial pathway in renal proximal tubular cells [55]. It is worth mentioning that mitochonic acid 5 enhances ATP production by facilitating ATP synthase oligomerization and supercomplex formation with mitochondrial protein mitofilin independent of OXPHOS and activity of complexes I–IV. Mitochonic acid 5 increases ATP production and cell viability of fibroblasts from patients with mitochondrial disease [56,57], improves renal function in the ischemia-reperfusion or cisplatin-induced AKI injury mouse and prolongs the life span of mice with mitochondrial disease [58]. The evidence shows that the modulation of mitochondrial energy metabolism is essential to cellular metabolism and renal function.

### 2.4. Improvement of Mitochondrial Biogenesis

**Figure 5:**
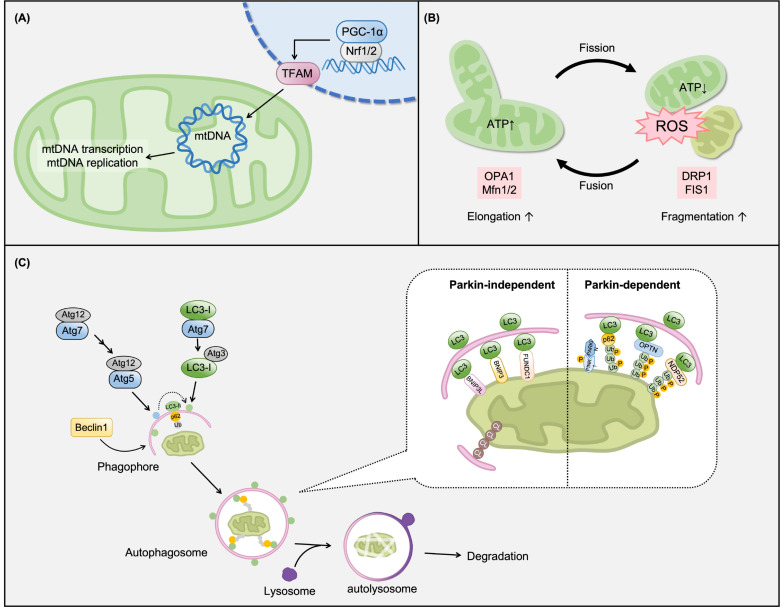
Molecular mechanisms of mitochondrial turnover. (**A**) Mitochondrial biogenesis. Nuclear peroxisome proliferator-activated receptor-gamma coactivator 1-alpha (PGC-1α) activates its target genes Nrf1/2. Transcription factor A, mitochondrial (TFAM) promotes mtDNA transcription and replication to generate new mitochondria. (**B**) Mitochondrial fusion/fission. Mitochondria are highly dynamic organelles that frequently fuse and divide. Mitochondrial fusion is mediated by mitofusins (Mfns) and optic atrophy protein 1 (OPA1). Mitochondrial fission is mediated by dynamin-related proteins and mitochondrial fission 1 protein (FIS1). (**C**) Mitophagy. Mitochondrial quality control systems are essential for the maintenance of functional mitochondria. Mitophagy is the selective degradation of mitochondria by autophagy. In mitophagy, the target mitochondria are recognized by the autophagosomes and delivered to the lysosome for degradation.

Mitochondrial abnormalities may be caused by oxidative stress or inflammation in metabolic disorders [3]. Mitochondrial biogenesis and mitochondrial DNA (mtDNA) maintenance are important for new mitochondrial generation and energy supply [59]. Biosynthesis of mitochondrial proteins is regulated by the nucleus and mitochondria, and most factors are encoded by nuclear genes and synthesized outside of the mitochondria [59]. The transcriptional coactivator peroxisome proliferator-activated receptor-gamma coactivator 1-alpha (PGC-1α) acts as a major regulator to control various signaling cascades, including Nrf1/2 and transcription factor A, mitochondrial (TFAM), which are involved in mitochondrial biogenesis for mitochondrial dynamics and energetics to maintain homeostasis (Figure 5A) [60,61,62]. The expression of PGC-1α has been demonstrated to be decreased in experimental CKD models and in the kidneys of CKD patients, most notably those with diabetic nephropathy [63,64].

Moreover, PGC1α protects human renal tubule cells from H_2_O_2_-mediated apoptotic injury by upregulating Nrf2 via glycogen synthase kinase 3β (GSK3β) inactivation [65], which is a key regulator of ROS-dependent cell death [66]. In contrast, serum- and glucocorticoid-induced kinase 1 (SGK1) is required for mitochondrial biogenesis signaling. Inhibition of SGK1 is much more sensitive to H_2_O_2_-induced toxicity in renal proximal tubular cells. Furthermore, SGK1 overexpression showed a protective effect in H_2_O_2_-induced oxidative injury cells by inactivating GSK3β [67]. The findings indicate that a mitochondria-targeted therapeutic strategy for the PGC1α-mediated pathway would be useful for improving oxidative damage in the kidney.

The polyphenol of grape seed, grape seed procyanidin B2, can significantly increase the viability of renal mesangial cells exposed to oxidative stress by elevating the antioxidative enzymes activity of glutathione peroxidase and SOD, as well as by enhancing the PGC-1α-mediated pathway and mtDNA content [68]. Curcumin has been demonstrated to have anti-inflammatory and antioxidant activities for the improvement of patients with CKD or CKD model [69]. Pretreatment with curcumin also decreases 5/6Nx-induced alterations early in mitochondrial dynamics, bioenergetics, and oxidative stress, which may be associated with the preservation of renal function [70]. In addition, in UUO models the anti-fibrotic drug fluorofenidone [71] and an isothiocyanate sulforaphane [72] also improved mitochondrial biogenesis by increasing the PGC-1α-mediated pathway. These data show that oxidative stress causes severe mitochondrial damage; however, mitochondrial biogenesis regulates new mitochondrial generation for mitochondrial homeostasis and energy metabolism to protect renal cells from oxidative injury.

### 2.5. Maintaining Mitochondrial Dynamics (Fusion/Fission and Mitophagy)

To control the normal function of mitochondria, they undergo competing sustainable processes: compensation of dysfunction by fusion, and elimination of damage by fission and mitophagy. Mitochondrial oxidative stress causes an imbalance in mitochondrial fusion/fission and results in mitochondrial fragmentation, which induces mitochondrial dysfunction and apoptotic cell death (Figure 5B) [73,74]. Optic atrophy-1 (OPA1) and Mfn1/2 are fusion-related proteins, and DRP1 and FIS1 are fission-related proteins. All are considered core proteins that modulate mitochondrial dynamics [75,76]. A study isolated the mitochondria from the kidneys of 5/6Nx CKD model and found a decrease in OPA1 and Mfn1, as well as increased expressions of FIS1 and DRP1 in the mitochondria [70]. Mfn2 deficiency in the kidney causes mitochondrial fragmentation without affecting renal tubular function during development or under non-stress conditions. However, Mfn2 deficiency exacerbates renal epithelial cell injury by promoting mitochondria-mediated apoptosis [77]. In contrast, Mfn2 overexpression inhibits apoptosis and ROS production and prevents mitochondrial dysfunction in renal cells [78]. In addition, calcitonin is a peptide hormone produced and released mainly in the thyroid gland for calcium homeostasis in blood [79]. Mitochondrial fragmentation was followed by an increase in DRP1 and FIS1 expression in the cerebral cortex of adenine-induced CKD mice with hypercalcemia; however, calcitonin inhibited the development of hypercalcemia and suppressed DRP1/FIS1-mediated mitochondrial fragmentation to attenuate hypercalcemia induced injury [79].

Further, to control and maintain mitochondria quality, damaged mitochondria are degraded by selective autophagy of mitochondria, which is termed mitophagy [80,81]. Increasing evidence suggests that oxidative stress acts as the main intracellular signal transducer to sustain autophagy. As shown in Figure 5C, in mitophagy, damaged mitochondria are engulfed by double-membrane vesicles called autophagosomes and degraded through the action of lysosomes contained within the vesicles. In this complex process, the LC3-II/I ratio and p62 protein are central autophagy-related factors that are ubiquitously used as autophagy markers to measure autophagic activity [82]. p62 binds directly to LC3 to deliver selective autophagic cargo for degradation via autophagy [83]. For elimination of dysfunctional mitochondria, the pathway can be roughly divided into Parkin-dependent and Parkin-independent mitophagy (Figure 5C). Phosphatase and tensin homolog-induced putative kinase 1 (PINK1) functions is an initiation protein as a signal-amplifying protein in the priming of mitochondria for mitophagy. Furthermore, Parkin-independent mitophagy, including B-cell lymphoma 2 interacting protein 3 (BNIP3), BNIP3L, and FUN14 domain containing 1 (FUNDC1) receptors, are located in the outer mitochondrial membrane (OMM) to trigger mitophagy [84,85,86,87]. The modulation of mitophagy is considered a possible therapeutic approach for renal injury [88]. Several studies have reported an increase in autophagic activity in kidney injury models. In UUO, autophagy in proximal tubular cells is enhanced and mitochondrial structure and content are altered. Enhanced autophagy is evidenced by increased expression of Beclin1, autophagy-related 5 (Atg-5)-Atg12, and LC3 in kidney tissue in UUO [22]. Gene Ontology and Kyoto Encyclopedia of Genes and Genomes pathway analyses revealed significant mitochondrial impairment, including decreased matrix metalloproteinase (MMP) and imbalanced mitochondrial dynamics, excessive oxidative stress, and activation of Pink1/Parkin-mediated mitophagy in UUO rats [89]. Similarly, mitochondrial injury was evident by enhanced mitophagy via the regulation of Beclin1, Park2, LC3β, and p62 levels in H_2_O_2_-induced proximal tubular epithelial cells [90,91]. However, the protective role of macrophage mitophagy during kidney fibrosis has also been reported in UUO- and adenine diet-induced renal injury models. In one study, inhibition of macrophage mitophagy prevented kidney fibrosis by regulating the Pink1/Parkin-mediated pathway [88]. Mitochondrial turnover is essential for the maintenance of healthy mitochondria, especially in the kidney, owing to its metabolic response [92].

Sulforaphane is an antioxidant that significantly increases nuclear Nrf2 translocation and decreases mitochondrial Bax translocation and cyt c release, which protect kidneys from UUO-induced renal oxidative stress, autophagy, and mitochondria-mediated apoptosis [72]. Tongluo Yishen decoction is a herbal-based traditional medicine that has been extensively used to treat CKD in China [89]. The decoction improves mitochondrial dynamics and alleviates mitophagy clearance deficiency, thereby protecting renal function [89]. It suggests that oxidative stress causes an imbalance in mitochondrial fusion/fission and results in mitochondrial fragmentation, in which abnormal mitochondria aggravate oxidative injury and excessive mitophagy. The regulation of mitochondrial fusion/fission and mitophagy protects cell from oxidative stress-induced cell death.

### 2.6. Others

#### 2.6.1. Cardiolipin

Cardiolipin is a tetra-acyl anionic phospholipid containing a central prochiral carbon, two phosphates, and four acyl chains in the IMM [93]. Cardiolipin plays a central role in many reactions and processes involved in mitochondrial function and dynamics [94]. Cardiolipin is essential for the formation of mitochondrial cristae and organizes the components of the ETC, ATP production, mitochondrial biogenesis, mitophagy, and cellular apoptosis [95,96]. An early review indicated that cardiolipin peroxidation and mitochondrial dysfunction were observed in various diseases, including heart ischemia/reperfusion, non-alcoholic fatty liver disease and diabetes [97]. In any case, renal lipid peroxidation has been observed in renal injury, which may contribute to mitochondrial injury by increasing cardiolipin peroxidation and decreasing cardiolipin content [96]. Moreover, a recent review summarized studies on cardiolipin, lipid peroxidation, and renal function, revealing that peroxidation or loss of cardiolipin is associated with the progression of acute kidney injury and CKD [98].

SS-31 is a cell-permeable peptide that selectively targets cardiolipin and modulates its interaction with cyt c [96,99]. The SS-31/cardiolipin complex reportedly inhibited cardiolipin peroxidation, protected cristae membranes during renal injury, and restored mitochondrial bioenergetics to prevent mitochondrial swelling in both endothelial and epithelial cells [96,99,100]. The data suggest that mitochondria-specific proteins or lipids can also be considered as a key target for mitochondrial homeostasis.

#### 2.6.2. Mitochondria-Mediated Apoptotic Pathway

Excessive production of ROS promotes mitochondria-mediated apoptosis, which plays a central role in cell homeostasis and the immune system [5,6]. OMM permeabilization is regulated by the Bcl-2 family of proteins, and the mitochondrial protein cyt c is subsequently released into the cytosol to activate caspases and initiate apoptotic cell death [101,102].

Neutral *Morchella conica* polysaccharide-2 is an antioxidant with a much stronger chelating ability for ferrous ions and a higher ability to scavenge free radicals [103]. Polysaccharide-2 dose-dependently preserved the cell viability of H_2_O_2_-induced human embryonic kidney cells, reduced ROS generation and increased MMP levels, significantly downregulated protein expressions of Bax and cleaved caspase 3, and significantly upregulated Bcl-2 levels [103]. Carnosine is also an antioxidant that decreases NOX4 expression and ROS production and increases SOD activity [104]. These activities result in reduced oxidative stress and protect cells from mitochondrial-mediated apoptotic cell death.

## 3. Causes and Risk Factors of Oxidative Stress-Related Mitochondrial Dysfunction in CKD and ESRD

The kidney is the most sensitive target organ for various toxins, and may be related to the progression of CKD and ESRD, with increased morbidity and mortality. The following subsections describe common environmental- and lifestyle-related toxins associated with kidney diseases.

### 3.1. Environmental Renal Injury

The kidney has been established as the main target organ for several types of environmental pollution, including ESRD, and is associated with increased morbidity and mortality. The studies discussed below relate to environmental pollution, oxidative stress, and renal mitochondrial injury.

#### 3.1.1. Air Pollution

Vehicle exhaust consists of pro-oxidants that include CO, CO_2_, NO_2_, and small particulate matter (PM). These are public health hazards and may be related to the risk of CKD. Kidney damage has been observed in humans and animals exposed to PM [105]. PM increases mtROS levels and reportedly decreases MMP in proximal tubular cells, inducing autophagy and leading to apoptotic cell death [105]. Moreover, rats exposed to simulated vehicle exhaust, containing 13% CO_2_, 0.1% NO_2_ and 0.68% CO in air, displayed an increase in oxidative stress, and decreased antioxidant activity in the kidney [106]. Mitochondrial dysfunction was also observed, with enhanced mitophagy and reduced fusion, resulting in low ATP production, complex IV, and ATP synthase levels [106].

#### 3.1.2. Heavy Metals

Environmental and industrial pollutants are associated with various health problems. Increasing evidence indicates that certain heavy metals have highly toxic and carcinogenic effects in humans and animals. The kidneys are the main deposition sites for toxic metals. Cadmium (Cd) [107,108], chromium (Cr) [109], lead (Pb) [110], molybdenum (Mo) [111,112], uranium (U) [113,114], and tungsten (W) [115] are common toxic metals that have been reported to cause severe oxidative stress by increasing ROS production, decreasing the activity of antioxidant enzymes, including SOD, glutathione peroxidase, catalase, glutathione S-transferase, and glutathione, and inhibiting the activities of ATPase in the kidney. These common toxic metals might act on the mitochondrial apoptosis pathway and decrease MMP and mitochondrial ETC activity, resulting in mitochondrial swelling [107,108,109,110,111,112,113,114,115]. A study also indicated that co-exposure to Mo and Cd caused oxidative damage in duck renal tubular epithelial cells, with a synergistic effect of the two metals [111]. Mo enhanced autophagy and caused cell injury in duck renal tubular epithelial cells; however, using an autophagy inhibitor, 3-methyladenine, aggravated mitochondrial dysfunction by regulating oxidative stress in Mo-induced cells [112]. Furthermore, aluminum induces apoptosis and activates PINK1/Parkin-mediated mitophagy in the kidneys of mice, which causes severe renal injury in the kidneys of Parkin-deficient mice [116]. These studies indicate that the regulation of mitochondrial function may be helpful for renal redox conditions and may promote kidney health against heavy metals.

Natural antioxidants, including α-lipoic acid [108], caffeic acid phenethyl ester [117], p-coumaric acid [118], *Potentilla anserina* polysaccharide [119], trehalose [120], and catechin [121], improve mitochondrial redox homeostasis and ameliorate the changes and injury caused by Cd. Resveratrol is a well-known antioxidant and popular ingredient in dietary supplements that ameliorates Cd-induced mitochondrial dysfunction by enhancing mitochondrial biogenesis associated with the upregulation of Sirt1/3, PGC-1α, Nrf1, and TFAM. Resveratrol can attenuate the Cd-induced imbalance in mitochondrial fusion and fission, which reverses PINK1/Parkin-mediated mitophagy [122]. Pretreatment with selenium was shown to partially block Cd-induced injury, including ROS generation, MMP collapse, cyt c release, and mitochondrial apoptotic pathways [123]. Selenium-deficient mouse podocytes and renal tubular epithelial cells show increased oxidative stress. Complex IV and cyt c levels were downregulated, while Sirt1 and PGC-1α levels were increased in selenium-deficient mice [124]. These findings suggest that selenium deficiency may result in oxidative damage to the mitochondria and kidneys [123,124].

However, other studies have shown the protective effects of several components in renal cells induced by other toxic metals. Pretreatment with carvedilol, a drug for hypertension and heart failure treatment, restored renal tissue antioxidant and mitochondrial respiratory enzyme activities, and decreased the elevated lipid peroxidation in rats with Cr-induced nephrotoxicity [109]. Puerarin is a potent ROS scavenger that protects renal cells from Pb-induced oxidative stress by inhibiting oxidative stress and lipid peroxidation, thereby increasing antioxidant enzyme activity and inhibiting apoptosis [110,125]. Depletion of MMP and caspase-3 activity were markedly inhibited by puerarin in the kidneys of Pb-treated rats [110] and primary rat proximal tubular cells [125]. *Polygonatum kingianum* is a well-known functional food that contains polysaccharides, saponins, flavonoids, and phenols. A study reported that *Polygonatum kingianum* ameliorated U-induced nephrotoxicity by regulating the GSK3β/Nrf2 antioxidant pathway [126].

#### 3.1.3. Fungicides, Herbicides, and Insecticides

Kresoxim-methyl and thioacetamide are fungicides that cause renal injury. Kresoxim-methyl causes renal injury by inhibiting complex III and decreasing antioxidant defense, leading to mitochondrial dysfunction with an elevated production of mitochondrial O_2_^−^ and decreased MMP [127]. Thioacetamide-induced renal oxidative stress decreases mitochondrial biogenesis, and increases autophagy and inflammatory, apoptotic, and fibrotic responses [128]. Platelet-rich plasma has progressively gained attention for its biological activity. Platelet-rich plasma increases the expressions of PGC1α and Beclin1 to protect cells against thioacetamide-induced renal injury by alleviating oxidative stress and improving mitochondrial biogenesis and autophagy [128]. Atrazine is a herbicide that induces renal damage by causing a decrease in mitochondrial cristae, mitochondrial swelling, mitochondrial dysfunction, and increased oxidative stress, modulating the Nrf2 signaling pathway [129]. Deltamethrin is an effective insecticide that causes an increase in intracellular ROS accompanied by elevated p66shc phosphorylation at Ser36 and translocation of p66shc from the cytoplasm to the mitochondria. p66shc mediated delatamethrin-induced oxidative stress may be partly responsible for its toxic effects [130]. Permethrin is an insecticide that induces oxidative stress by increasing lipid peroxidation and protein oxidation, and decreasing antioxidant defense in the mitochondria of kidney tissue [131]. The ethyl acetate fraction extracted from *Fumaria officinalis* improved the antioxidant status and mitochondrial bioenergetics in permethrin-treated rats [131].

#### 3.1.4. Plasticizer Compounds/Organic Pollutants

Plasticizers, such as bisphenol A [132] and di-(2-ethylhexyl) phthalate [133,134], are environmental pollutants that cause renal damage. These plasticizers may induce pro-inflammatory cytokines, increase lipid peroxidation, and decrease antioxidant defense [132,133,134]. These plasticizers also cause mitochondrial oxidative damage, as evidenced by ATP depletion and MMP collapse in renal proximal tubular cells [132,133,134] and rat kidney [134]. As oxidative stress triggers antioxidant defense to protect cells, bisphenol A also induces Nrf2-mediated antioxidant responses [132]. Conversely, a study revealed that di-(2-ethylhexyl) phthalate decreased total Nrf2 levels; however, the authors did not mention Nrf2 nuclear translocation [135]. Moreover, bisphenol A-induced injury is associated with aberrant mitochondrial dynamics [136]. Increased mtROS, disrupted MMP, swelling, and impaired mitochondrial fission caused by bisphenol A have been mitigated by N-acetyl-cysteine (NAC). NAC enhances AMPK-PGC-1α-SIRT3 signaling protein expression, which increases the expression of MnSOD in bisphenol A-treated renal cells [137,138]. As expected, intracellular glutathione content can be elevated by NAC, and DNA damage induced by di-(2-ethylhexyl) phthalate is almost completely abolished by NAC [133]. Moreover, melatonin [139], quercetin [140], and astaxanthin [141] diminished mitochondrial oxidative stress and maintained mitochondrial function against bisphenol A nephrotoxicity.

Organic pollutants are categorized as probable carcinogens and bioaccumulate in fatty tissues of animals and humans, reaching toxic levels upon continued exposure. Bromobenzene is used as an additive in motor oils as a crystallizing solvent. Withaferin A, isolated from *Withania somnifera*, has recently become an attractive phytochemical for preclinical studies on various diseases [142,143]. Withaferin A improved bromobenzene-induced nephrotoxicity and mitochondrial dysfunction in rats [142] and mice [143] by improving the decrease in the activities of mitochondrial enzymes and imbalance in Bax/Bcl-2 expression in the kidneys.

#### 3.1.5. Nanoparticles

Multi-walled carbon nanotubes (MWCNTs) [144,145] and the green synthesis of metal nanoparticles [146,147] are techniques used in industry, agriculture, and drugs worldwide. However, their risks and safety for the human body should be considered. The possible hazards of MWCNTs and nanoparticles have recently become a major focus of research.

Studies evaluated the potential toxicity and general mechanisms involved in MWCNT-induced human embryonic kidney cells [144] and rat kidney mitochondria [145]. Exposure to MWCNTs resulted in cell membrane damage, increased formation of ROS and malonaldehyde, decreased intracellular glutathione levels, MMP collapse, and mitochondrial release of cyt c [144,145]. The cytotoxicity of MWCNT has been associated with increased oxidative stress and mitochondrial damage. The advantages of metallic nanoparticles, such as their small size, high reactivity, and great capacity, could become potentially lethal factors by inducing adverse cellular toxic and harmful effects, which are unusual in their micron-sized counterparts [145]. Furthermore, nanoparticles can enter organisms during ingestion or inhalation and can translocate within the body to various organs and tissues where the nanoparticles react toxically [146,147]. Gold nanoparticles (AuNPs) are widely used for biomedical applications, and the toxicity of AuNPs has been described in kidney tissue with a decrease in antioxidative enzymes activity and altered energy metabolism in the kidney, leading to oxidative damage [148]. Silver nanoparticles (AgNPs) caused significant damage and dysfunction in renal proximal tubular cells and renal mitochondria [149]. Exposure to copper nanoparticles (CuNPs) can induce oxidative stress in the kidney. In one study, CuNP exposure reciprocally regulated Bcl-2 family protein expression, disturbed MMP, and subsequently helped release cyt c from the mitochondria into the cytosol. CuNPs can trigger mitochondria-mediated apoptotic pathways in the kidneys against oxidative stress [150]. Platinum nanoparticles (PtNPs) also increased ROS levels and lipid peroxide in human embryonic kidney cells, with an increase in caspase 3 expression, decreased MMP, and DNA fragmentation [151]. These findings suggest the importance of mitochondria in nanoparticles-induced renal injury. However, oxidative stress and mitochondrial dysfunction induced by AuNPs can be ameliorated by NAC treatment [152]. This suggests that other antioxidants may also protect the cells from nanoparticles-induced damage by maintaining mitochondrial function.

Although nanoparticles may cause oxidative stress and mitochondrial dysfunction, one study suggested that rats exposed to iron oxide nanoparticles did not show altered mitochondrial ETC activity in the kidney and other organs [153]. Moreover, CuNPs elevated renal oxidative stress and damaged rat kidney or porcine kidney epithelial cells without causing mitochondrial injury [154]. There are studies discussed about biodistribution and clearance of AuNPs, indicating that the smaller size of the AuNPs is eliminated mainly through the kidneys rather than the hepatobiliary system [155,156]. Synthesized AuNPs of different sizes (13 nm and 60 nm) and shapes (spheres and stars) and coated with different materials (11-mercaptoundecanoic acid and sodium citrate) mainly targeted mitochondria and showed cytotoxicity in a dose-dependent manner. Both shapes showed more toxicity with a smaller size. However, the 13 nm sphere-shaped AuNPs (both 11-mercaptoundecanoic acid- and sodium citrate-coated) proved to be the most toxic among all types of AuNPs. They increased ROS production, decreased MMP and caused apoptosis in proximal tubular cells [157]. In vivo, there was no toxic effect on the kidneys of the Swiss albino mice after intraperitoneal injection of 50 nm AuNPs at a dose of 170 μg/kg bodyweight [158], whereas 50 nm AuNPs (22 μg/kg bodyweight) induced an acute phase increase in proinflammatory cytokines expression in Wistar-Kyoto rats after 24 h injection [159]. These findings suggest that the toxicity of nanoparticles may be related to their renal clearance due to their size and metal type. It is important to consider that the accumulation of nanoparticles in the kidneys can induce cell damage and kidney dysfunction. Concerns regarding nanoparticle toxicity in different species should be assessed individually due to the experimental models and characteristics of nanoparticles.

#### 3.1.6. Food Contamination

Several studies have suggested that food contamination is a risk factor for nephrotoxicity, and is correlated with impaired mitochondrial function. Acrylamide is naturally formed in foods cooked at high temperatures, such as potato products. A study involving rats exposed to acrylamide documented increased lipid peroxidation and protein carbonyl levels, and decreased mitochondrial antioxidant enzyme activities, mitochondrial metabolic function, and ATP levels [160]. However, argan oil can protected mitochondrial function in acrylamide-induced liver and kidney injury of rats [160]. The substance 3-Monochloropropane-1,2-diol is another food processing contaminant and carcinogenic agent that can induce nephrotoxicity. Increased levels of ROS and decreased levels of MMP and glutathione were documented in isolated rat renal cells and human embryonic kidney cells treated with 3-Monochloropropane-1,2-diol; the alterations were correlated with the impairment of mitochondrial OXPHOS, leading to the release of cyt c and induced apoptosis [161,162].

Aflatoxin B1, ochratoxin A, and patulin are common mycotoxins with various toxicological effects, especially nephrotoxicity [163,164,165]. Aflatoxin B1 induces kidney damage, oxidative stress, mitochondrial damage, apoptosis and activates Pink1/Parkin-mediated mitophagy [163]. Notably, a study comparing ochrtoxin A toxicity between sexes observed modulation of inflammation, proliferation, and oxidative stress in both sexes. However, cell damage, fibrosis, cell signaling, and metabolism were exclusively altered in males, whereas renal safety biomarkers and mitochondrial biogenesis were exclusively enriched in females. This may contribute to the high estrogen levels in females, and requires further investigation [164]. Patulin regulates the expression of genes and proteins involved in mitochondrial ETC, resulting in dysfunction of OXPHOS and induction of ROS overproduction. NAC improves patulin-induced apoptosis in human embryonic kidney cells by decreasing ROS, modulating ETC activity, and maintaining mitochondrial function [165].

Deoxynivalenol, zearalenone, and fumonisin B1 are among the most toxicologically important Fusarium toxins commonly found in nature that cause nephrotoxicity [166,167]. Glutathione reductase and total SOD activities were reportedly affected by deoxynivalenol, zearalenone, and fumonisin B1; the altered activities induced ROS and malonaldehyde production, increased apoptosis, and the regulation of the mRNA expression of Bax, Bcl-2, caspase-3, caspase-9, cyt c, and p53 [166]. In one study, deoxynivalenol can also activate the Nrf2 antioxidant pathway. Abnormal mitochondria were observed and the expression of the mitochondrial biogenesis-related factors voltage-dependent anion channel 1 and cyt c was upregulated by deoxynivalenol. Deoxynivalenol significantly reduced Sirt3 expression and induced mitochondrial fusion inhibition and mitophagy [167]. The same research group also suggested in another paper that NAC reduced oxidative damage and inhibited apoptosis induced by Fusarium toxins in porcine kidney cells [166].

### 3.2. Lifestyle-Related Renal Injury

#### 3.2.1. Chronic Alcohol/Ethanol Consumption

Chronic ethanol consumption induces renal mitochondrial protein hyperacetylation, which participates in the suppressed regulation of metabolic and mitochondrial processes, such as antioxidant defense and energy metabolism [168]. Acetylation of antioxidative enzymes, including MnSOD, glutathione reductase, and glutathione S-transferase, significantly increases ethanol toxicity. The results demonstrated increased protein acetylation concurrent with depleted glutathione, altered cysteine redox potential, and 4-hydroxynonenal protein modifications in an early stage of the alcoholic nephrotoxicity model [168]. This suggests that ethanol causes mitochondrial protein hyperacetylation, with the potential to impact mitochondrial metabolic and antioxidant processes.

#### 3.2.2. High Fat Diet (HFD)/Obesity/Metabolic Syndrome

Obesity [169,170,171,172,173] and metabolic syndrome [174,175] are independent risk factors for CKD and its progression to ESRD, even in the absence of diabetes or hyperglycemia. An HFD can induce glomerular hypertrophy, fibrosis, and scarring in the kidneys [169,171,172,173]. Decreased MnSOD activity and increased ROS levels can occur in the renal mitochondria without obvious damage to mitochondrial ETC function [169]. This may reflect adaptation in the kidneys of an HFD-treated mouse, to maintain respiratory function as a response to oxidative stress [169]. Moreover, increased oxidative stress suppresses the expression of long-chain acyl-CoA synthetase-1, the key enzyme in fatty acid oxidation by mitochondria, ultimately leading to renal lipid accumulation in renal tubules and accelerating the development of obesity-related nephropathy [170,171]. Effects of an HFD on renal oxidative stress, albuminuria, fibrosis, and podocyte loss/insulin resistance have been described [171]. Accumulated lipids also induce mitochondrial fission and apoptosis in renal cells, suggesting that consumption of an HFD causes renal cell injury, which may be attributed to oxidative stress and mitochondrial dysfunction, promoting excess programmed cell death [172]. Moreover, palmitic acid and oleic acid are fatty acids often used for investigating lipotoxicity [170,171,173,176]. ROS production and renal lipid deposition have been observed in palmitic acid-treated proximal tubular cells [170,171,173]. Oleic acid stimulates the promoter of p66shc and increases mtROS production, resulting in decreased MMP and cell injury in cultured renal proximal tubular cells [176]. Nrf2 is inhibited in obesity-related nephropathy, which results in increased ROS production and oxidative stress [171]. Regulation of the mitochondrial antioxidant system and fatty acid β-oxidation can be useful for reducing lipotoxicity in the kidneys.

Early periods of metabolic syndrome induce mitochondrial abnormalities and dysfunction in the kidney [174,175]. The leptin receptor-deficient POUND mouse shows high plasma uric acid levels and develops obesity and metabolic syndrome secondary to hyperphagia [174]. In the POUND mouse, the presence of metabolic syndrome was associated with earlier development of renal disease (12 months) and earlier mortality compared to healthy mice. After the administration of adenine, kidney disease was worse in POUND mice; this was associated with greater tubular injury with a decrease in kidney mitochondria, lower tissue ATP levels, and worse oxidative stress [174]. POUND mice with similar levels of renal function as adenine-treated wild-type mice also showed worse sarcopenia, with lower tissue ATP and intracellular phosphate levels [174]. These findings suggest that obesity and metabolic syndrome accelerate CKD progression, which is associated with abnormal energy metabolism and may be due to mitochondrial dysfunction.

In the kidney mitochondria, curcumin treatment was reported to significantly increase oxygen consumption and decrease lipid and protein peroxidation levels in HFD-induced obese mice [172]. In another study, silymarin significantly ameliorated HFD-induced glucose metabolic disorders, oxidative stress, and pathological alterations in mouse kidneys. Silymarin also significantly mitigated renal lipid accumulation, β-oxidation, and mitochondrial biogenesis in HFD mice and palmitic acid-treated proximal tubular cells [173]. Pigs with metabolic syndrome fed a high-cholesterol/carbohydrate diet showed decreased cardiolipin content, altered mitochondrial morphology and function, and increases of renal cyt c-induced apoptosis, oxidative stress, and tubular injury. Mitochondria-targeted peptide SS-31 restored mitochondrial structure and function, improved medullary oxygenation, and decreased renal injury in the pigs [175]. Chicoric acid has potential as a functional natural antioxidant, which was demonstrated to be capable of ameliorating metabolic disorders, including overweight, hyperglycemia, hyperlipidemia, and hyperuricemia in HFD-fed mice [177]. Alleviation of lipid accumulation and apoptosis was observed in palmitic acid-exposed renal proximal tubular cells. The authors reported that chicoric acid reduced mitochondrial damage and oxidative stress in the kidneys of HFD-fed mice and PA-treated renal proximal tubular cells, activated the Nrf2 pathway, increased PINK and Parkin expression, and regulated LC3, p62, Mfn2, and FIS1 expression. Finally, the authors demonstrated that chicoric acid improved mitochondrial dynamics and mitophagy to alleviate mitochondrial damage in renal tubular epithelial cells in HFD-induced CKD mice [177].

Conversely, dietary restriction or calorie restriction is reported to have beneficial effects for human health. These restrictions are considered efficient approaches to ameliorate the severity of various pathological conditions, including renal diseases. A study showed that renal oxidative injury was aggravated and mitophagy was markedly decreased in aging HFD kidneys, whereas they were markedly ameliorated in kidneys upon dietary restriction [178]. These results suggest that an HFD causes severe abnormal mitochondrial morphology and dysfunction, while antioxidants and dietary restriction protect mitochondria and reduce oxidative stress in obesity-related nephropathy.

#### 3.2.3. Smoking

Nicotine is a major tobacco alkaloid that links smoking with renal damage. Chronic nicotine exposure induces oxidative stress, as evidenced by increased levels of mitochondrial-derived ROS in renal proximal tubular cells [179,180]. Maternal cigarette smoke is also considered a risk factor for developing CKD in offspring. Increased mitochondrial oxidative damage at birth and in adulthood contributes to adverse effects of maternal smoking on renal disorders [180]. Abnormal mitochondrial structure along with a reduction in ETC levels and activities of glutathione peroxidase and MnSOD in the kidneys of pregnant mice exposed to cigarette smoke and their offspring have been documented. Moreover, mitochondrial damage caused by maternal smoking may play an important role in the development of CKD in adults [179,180]. The phosphorylation of serine36 of pro-apoptotic p66shc protein facilitates its mitochondrial translocation and cyt c binding. The binding generates oxidative stress that leads to the injury of renal proximal tubular cells during chronic nicotine exposure [181]. L-carnitine supplementation has been reported to mitigate the effects of maternal cigarette smoke exposure on renal pathology, renal oxidative stress, and mitochondrial density in mouse offspring [179]. Coenzyme Q10 strongly inhibited nicotine-mediated ROS production in one study [181]. Both nicotine and coenzyme Q10 stimulate the p66shc promoter, and nicotine exposure results in mitochondrial translocation of p66shc. CoQ10-stimulated and non-mitochondrial p66shc activates the antioxidant MnSOD to rescue cells from nicotine-induced oxidative stress and consequent apoptosis [181].

The studies indicated that renal failure and oxidative stress caused by these risk factors, due to mitochondrial antioxidants’ imbalance and subsequent mitochondrial swelling, caused damage to aggravate oxidative injury. However, the enhancement of antioxidants’ defense or improvement of mitochondrial enzymes activities can be a useful tool for improving oxidative stress-related renal failure (Table 1). Moreover, chronic inflammation contributes to the progression of CKD, and mitochondrial redox homeostasis has been associated with inflammation condition [182]. A recent review mentioned that “mito-inflammation” should be a new concept: that mtROS contribute to mito-inflammation in inflammatory-related diseases [182]. The evidence provides opportunities for ameliorating oxidative stress and inflammation in several diseases, including renal diseases.

## 4. Conclusions

The review includes studies published in the previous decade that address the mitochondrial-targeted therapeutics of kidney dysfunction. A number of components have recently become candidates for clinical intervention in kidney diseases. Evidence indicates that abnormal mitochondrial homeostasis plays a critical role in the progression of CKD. Mitochondrial networks affect their own function and also interact with other cellular organelles, such as the nucleus and endoplasmic reticulum. Renal mitochondrial dysfunction may also affect the function of different tissues, such as the heart and muscle. The metabolic systems of organs are closely related to living organisms. The elucidation of clinical effects is warranted to address the specific needs of patients with renal failure. Components that target mitochondria may also display organ-specific sensitivity or toxicity in animals or humans. Therefore, we must carefully consider the hazards and safety of the components rather than overestimating the benefits in laboratory research. A better understanding of mitochondrial physiological functions in the kidney under oxidative stress will provide new insights for the discovery of useful therapeutic strategies to ameliorate kidney injuries and other mitochondrial-related diseases.

## Figures and Tables

**Table 1:** The causes and risk factors of mitochondrial-related oxidative injury in the kidneys or renal cells. The treatments which can improve renal injury and antioxidant status and their protective effects on mitochondrial function were listed. ↑ indicates an increase or an up-regulation, ↓ indicates a decrease or a down-regulation in the list.

Causes and Risk Factors	Mechanisms	Model [Ref.]	Treatment [Ref.]	Effects on Mitochondria
***Environmental renal injury***				
Air pollution				
particulate matter	mtROS↑, MMP↓, autophagy↑, mitochondrial-related apoptosis↑	Sprague Dawley (SD) rats [105]; human kidney proximal tubular (HK-2) cells [105]	−	−
gaseous mixtures	MMP and ATP↓, mitochondrial respiration and fusion↓, mitophagy↑	SD rats [106]	−	−
Heavy metals				
cadmium (Cd)	mitochondrial swelling, MMP and ATPase↓, PGC-1α/Nrf2-related pathway↑, mitochondrial-related apoptosis↑	human embryonic kidney (HEK293) cells [107,119]; SD rats [108,120]; Wistar rats [117,118,121]; BALB/c mice [119]; ICR mice [123]; Hy-Line Variety White chickens [122]	α-lipoic acid [108]	improved mitochondrial swelling; inhibited mitochondrial-related apoptosis
			caffeic acid phenethyl ester [117]	improved mitochondrial swelling and dysfunction
			p-coumaric acid [118]	regulated gluconeogenic and glycolytic enzyme activities;enhanced TCA cycle and ETC enzyme activities
			*Potentilla anserina* [119]	regulated PGC-1α/Nrf2-related pathway; inhibited mitochondrial-related apoptosis
			trehalose [120]	inhibited mitochondrial-related apoptosis
			catechin [121]	improved renal function and mitochondrial antioxidant status
			resveratrol [122]	improved renal mitochondrial injury via regulation of mitochondrial biogenesis and dynamics
			selenium [123]	inhibited mitochondrial-related apoptosis
chromium (Cr)	ETC and antioxidative enzymes activities↓, mitochondrial-related apoptosis↑	SD rats [109]	carvedilol [109]	enhanced antioxidants and ETC enzyme activities and inhibited mitochondrial-related apoptosis
lead (Pb)	MMP↓, PI3K/Akt/eNOS pathway↓mitochondrial-related apoptosis↑	primary rat proximal tubular cells [125]; Wistar rats [110]	Puerarin [110,125]	improved mitochondrial injury and inhibited mitochondrial-related apoptosis
molybdenum (Mo)	intracellular [Ca2+]↑, MMP↓, ATPase activity↓, mitochondrial content↓, mitochondrial-related apoptosis↑	primary duck renal tubular epithelial cells [111,112]	3-methyladenine [111]	an autophagy inhibitor;aggravated Mo-induced mitochondrial dysfunction by regulating oxidative stress
uranium (U)	mitochondrial swelling, mtROS and mtMDA↑, mtGSH, MMP, and ATP↓, ETC and ATPase activities↓, mitochondrial-related apoptosis↑	HK-2 cells [114,126]; Wistar rats [113]	*Polygonatum kingianum* [126]	improved mitochondrial injury and inhibited mitochondrial-related apoptosis via regulating the GSK3β/Nrf2-related pathway
tungsten (W)	mitochondrial swelling, mtROS and mtMDA↑, mtGSH, MMP, and ATP↓, mitochondrial-related apoptosis↑	Wistar rats [115]	-	-
aluminum (Al)	MMP↓, mitophagy↑, mitochondrial-related apoptosis↑	C57BL/6 mice [116]	-	Parkin deficiency aggravated Al-induced oxidative stress and mitochondrial damage
Fungicides, herbicides, and insecticides				
Kresoxim-methyl	intracellular [Ca2+] and mtROS↑, MMP↓	monkey kidney Vero CCL-81 cells [127]	−	−
thioacetamide	mitochondrial biogenesis↓, autophagy↑, mitochondrial-related apoptosis↑	albino rats [128]	platelet-rich plasma [128]	improved mitochondrial injury via regulating the PGC1α-related pathway; inhibited autophagy and mitochondrial-related apoptosis
Atrazine	mitochondria content↓ and damage↑,mitochondrial-related apoptosis↑	quail [129]	−	−
Deltamethrin	kidney and other organs failure	SD rats [130]	−	−
Permethrin	mitochondrial swelling, ETC activities↓	albino Wistar rats [131]	*Fumaria officinalis* extract [131]	improved renal injury, antioxidant status and mitochondrial bioenergetics
Plasticizer compounds/organic pollutants				
bisphenol A	intracellular [Ca2+] and mtROS↑, MMP and ATP↓, AMPK-PGC-1α-SIRT3-pathway↓, mitochondrial fission↑, mitochondrial-related apoptosis↑	HK-2 cells [132]; Wistar rats [136,137,138,139,140,141]	NAC [136,137,138]	inhibited mitochondrial fission; regulated AMPK-PGC-1α-SIRT3 signaling
			melatonin [139]	improved renal mitochondrial swelling and injury
			quercetin [140]	improved renal mitochondrial injury
			astaxanthin [141]	regulated ETC activities; inhibited mitochondrial-related apoptosis
di-(2-ethylhexyl) phthalate	mitochondrial swelling, MMP↓, mitochondrial content↓, mitochondrial oxidative stress↑, mitochondrial-related apoptosis↑	HEK293 cells [133,134,135]; Wistar rats [134]	NAC [133,135]	improved renal mitochondrial injury
bromobenzene	mtGSH↓, TCA cycle enzymes and ETC activities ↓, mitochondrial-related apoptosis↑	albino Wistar rats [142]; albino Swiss mice [143]	Withaferin A [142,143]	improved renal mitochondrial injury and mitochondrial enzymes activities; inhibited mitochondrial-related apoptosis
Nanoparticles				
multi-walled carbon nanotubes	mitochondrial swelling, SDH activity and MMP↓; mtROS↑, mitochondrial-related apoptosis↑	HEK293 cells [144]; Wistar rats [145]	Apigenin [145]	improved renal mitochondrial injury and mitochondrial enzymes activities; inhibited mitochondrial-related apoptosis
AuNPs	energy metabolism (ETC activity) impairment, MMP and ATP↓, mitochondrial-related apoptosis↑	HK-2 cells [152,157]; Wistar rats [148]	NAC [152]	improved mitochondrial injury and energy metabolism;inhibited mitochondrial-related apoptosis
AgNPs	mitochondrial swelling, mitochondrial enzyme activities↓	albino Wistar rats [149]; pig kidney epithelial LLC PK1 cells [149]	−	−
CuNPs	MMP↓, mitochondrial-related apoptosis↑	albino Swiss mice [150]	−	−
PtNPs	MMP↓, mitochondrial-related apoptosis↑	HEK293 cells [151]	−	−
Food contamination				
Acrylamide	ATP↓, mitochondrial enzyme activities↓	SD rats [160]	Argan oil [160]	improved mitochondrial enzymes activities
3-monochloropropane-1,2-diol	MMP and mtDNA↓, mitochondrial biogenesis↓, mitochondrial-related apoptosis↑	HEK293 cells [161]; C57 mice [162]; Wistar rats [161]	−	−
Aflatoxin B1	MMP and ATP↓, mitophagy↑, mitochondrial-related apoptosis↑	C57BL/6N mice [163]	−	−
ochratoxin A	males: cell damage, fibrosis, cell signaling, and metabolism↑;females: renal safety biomarkers and mitochondrial biogenesis↑	Fischer 344 rats [164]	−	−
patulin	ATP and MMP↓, ETC impairmentmitochondrial-related apoptosis↑	HEK293 cells [165]	NAC [165]	inhibited mitochondrial-related apoptosis modulated ETC activity, and maintaining mitochondrial function
deoxynivalenol, zearalenone, and fumonisin B1	mitochondrial swelling, mitochondrial biogenesis↓, fusion↓/fission↑, mitophagy↑, mitochondrial-related apoptosis↑	porcine kidney PK15 cells [166]; piglets (Duroc × Landrace × Yorkshire) [167]	NAC [166]	inhibited mitochondrial-related apoptosis
			*Lactobacillus rhamnosus* GG [167]	increased Sirt3 to maintain redox balance, regulated mitochondrial fusion/fission, and prevented mitophagy
***Lifestyle-related renal injury***				
Chronic alcohol				
	mitochondrial proteins acetylation↑	C57BL/6J mice [168]	−	−
HFD/obesity				
HFD-induced obesity/fatty acid-induced lipotoxicity	mitochondrial swelling, mitochondrial bioenergetic adaptation: PGC-1β, NRF2, TFAM, and ERRα ↑, mitophagy↑, ATP and MMP↓, oxygen consumption↓	HK-2 cells [170,171,173]; mesangial SV40 MES 13 cells [171]; mouse kidney proximal tubular TKPTS cells [176]; C57BL mice [169,171,172,173]; aged Fischer 344 rats [178];	silymarin [173]	regulated β-oxidation, and mitochondrial biogenesis
			NAC [171,176]	p66shc↓, MMP↓, mitochondrial fission↓mitochondrial-related apoptosis↓
			curcumin [172]	increased oxygen consumption; decrease lipid and protein peroxidation
			calorie restriction [178]	aggravated and mitophagy was markedly decreased in aging HFD kidneys, whereas they were markedly ameliorated
obesity patients/genic obesity animals	renal biopsy: ACSL1 and Nrf2↓ob/ob mice: ACSL1, Nrf2, and SOD↓; ROS and MDA↑	obesity-related nephropathy patients [170]; C57BL/6 J ob/ob mice [170];	−	−
Metabolic syndrome				
genic model	(with adenine diet) mtDNA and ATP↓, mitochondrial genes↓	POUND mice [174]	−	−
high-cholesterol/carbohydrate diet	cardiolipin content↓, cardiolipin remodeling↓, ATP↓, mitochondrial-related apoptosis↑	farm pig [175]	SS-31 [175]	improved renal mitochondrial cardiolipin content and ATP level; inhibited mitochondrial-related apoptosis
Smoking				
mother/offspring	mitochondrial density and mtDNA↑, p66shc↑, mtROS↑, ETC↓,mitochondrial-related apoptosis↑	rat kidney proximal tubular NRK52E2 cells [181]; Balb/c mice and offspring [179,180]	L-Carnitine [179]	improved renal mitochondrial respiration and reduced mtROS
			CoQ10 [181]	reduced ROS production via regulating p66shc-related pathway; inhibited apoptosis

## Abbreviations

5/6Nx	five-sixths nephrectomy
Atg	autophagy-related protein
ATP	adenosine triphosphate
BNIP3	BCL2 interacting protein 3
BNIP3L	BCL2 interacting protein 3 like
Cd	cadmium
CKD	chronic kidney disease
Cr	chromium
cyt c	cytochrome c
ESRD	end-stage renal disease
ETC	electron transport chain
FIS	mitochondrial fission 1
FUNDC1	FUN14 domain containing protein 1
GSK3β	glycogen synthase kinase 3 beta
H_2_O_2_	hydrogen peroxide
HFD	high fat diet
IMM	inner mitochondrial membrane
LC3	light chain 3
Mfn1/2	mitofusin 1/2
MitoQ	mitoquinone (10-(6′-ubiquinonyl)decyltriphenylphosphonium bromide)
MMP	mitochondrial membrane potential
MnSOD	manganese-superoxide dismutase
Mo	molybdenum
mPTP	mitochondrial permeability transition pore
mtDNA	mitochondrial DNA
mtROS	mitochondrial ROS
MWCNTs	multi-walled carbon nanotubes
NAC	N-acetyl-cysteine
NOX	NADPH oxidase
Nrf1/2	nuclear factor erythroid 2-related factor 1/2
O_2_˙^−^	superoxide
OMM	outer mitochondrial membranes
OPA1	optic atrophy 1
OXPHOS	oxidative phosphorylation
Pb	lead
PGC-1α	peroxisome proliferator-activated receptor-gamma coactivator-1alpha
ROS	reactive oxygen species
SGK1	serum- and glucocorticoid-induced kinase 1
Sirt1/3	silent mating type information regulation 2 homolog 1/3, sirtuin 1/3
SOD	superoxide dismutase
TFAM	mitochondrial transcription factor A
U	uranium
UCP2	uncoupling protein 2
UUO	unilateral ureteral obstruction
W	tungsten
